# Key Determinants of COVID-19 Vaccination Take-Up in Remote Rural Areas: Evidence From Colombia

**DOI:** 10.3389/ijph.2024.1606689

**Published:** 2024-06-13

**Authors:** Natalia Cantet, Marcela Ibañez, Juan Carlos Muñoz-Mora, Laura Maria Quintero

**Affiliations:** ^1^ Darla Moore School of Business, University of South Carolina, Columbia, SC, United States; ^2^ Georg-August-Universität Göttingen, Göttingen, Germany; ^3^ Escuela de Finanzas, Economía y Politica, Universidad EAFIT, Medellín, Colombia

**Keywords:** COVID-19 vaccination, perception of disease risk, hesitancy, health, Public institutions

## Abstract

**Objetives:**

The adoption of vaccines was a crucial factor in overcoming the COVID-19 pandemic. However, vaccination rates between rural and urban areas varied greatly. In this paper, our objective is to understand the individual and institutional factors associated with the uptake of vaccines in remote rural areas in Colombia.

**Methods:**

We interviewed a random sample of 800 households (1,592 individuals) in remote rural areas of Antioquia (Colombia) during February 2022 when vaccinations were available. Then, we use a linear probability model to explain the uptake of the COVID-19 vaccine.

**Results:**

The results indicate that the probability of having at least the first dose of the COVID-19 vaccine is positively associated with access to information, trust in police and army, and the perceived risk of contracting COVID-19. Trust in the church is negatively related to vaccination.

**Conclusion:**

Institutions can play a critical role in the management of pandemics. Timely information on the risks associated with the disease and perceived riskiness are key factors that mobilize the population to take the COVID-19 vaccine.

## Introduction

On 30 January 2020, the World Health Organization declared COVID-19 a public health emergency, launching a race to discover an effective vaccine to control the spread of the virus and reduce the mortality rate [1, 2]. Only 10 months later, the first vaccine had been developed, and the speed of this vaccine development has never been greater in world history [3]. Vaccination campaigns were essential to survive the pandemic and restore everyday life. In many developing countries, these campaigns exposed the structurally uneven access to health services between rural and urban areas. Recent research shows that COVID-19 hit rural areas relatively harder than urban areas in terms of socioeconomic conditions [4, 5]. However, few studies have focused on the consequences of COVID-19 in rural areas and how living in those areas could determine access to or availability of COVID-19 vaccines, especially in developing countries [6].

According to the Economic Commission of the United Nations for Latin America and the Caribbean (CEPAL) (2022), as of 15 July 2021, approximately 78.4% of the US population had received at least one dose of the COVID-19 vaccine. In Latin America and the Caribbean, the vaccination rate varied from country to country; the highest rates are in Chile and Cuba, with approximately 91% of Chileans and 88% of Cubans fully vaccinated [7]. In Colombia, approximately 82.5% had received at least one dose and approximately 70.5% were fully vaccinated [7]. In Antioquia, Colombia, where nearly 20% of the population lives in rural areas (1.4 million people), according to the 6 April 2021, report issued by [8], 4,907,392 first-dose vaccines had been administered (this is equivalent to 72 percent of the population), and 3,987,499 second-dose vaccines (58 percent of the population). However, there is limited evidence on implementing the program in rural areas and individual factors that affect the acceptability of the COVID-19 vaccine.

There are several barriers to implementing large vaccination campaigns in rural areas. The first barrier is the low capacity. Compared to urban areas, rural areas have fewer health facilities and fewer health resources, increasing their vulnerability to infections such as COVID-19 [4]. Even in developed nations, the situation is dire. For example, more than 4.7 million people in the United States live in 460 rural counties without hospital beds for general medical or surgical purposes, and around 16.4 million live without an intensive care unit (ICU) nearby [4, 5]. This panorama is even worse when you look at developing countries. In rural India, there are only 3.2 public hospital beds per 10,000 people, which makes the healthcare system neither adequate nor prepared to handle massive emergencies [9].

A second barrier relates to the remoteness and low density of rural areas. The primary road network is deteriorated and most municipal roads are unpaved. Access to broadband connectivity is also limited, with only 28.8 percent of rural households having broadband access. Few services are offered using mobile technologies [10, 11]. The situation of public order and violence is another factor limiting the implementation of the COVID-19 vaccine program in Colombia [12]. Armed conflict restricts access to healthcare for the rural population [13].

However, the low number of vaccines administered in rural areas cannot only be explained by availability. The willingness to get vaccinated is another important obstacle among rural households [14]. Although the US mortality rates from COVID-19 are higher in rural areas than in urban areas, on 11 August 2021, only 45.8% of adults in rural counties had been fully vaccinated, compared to 59.8% in urban counties [5]. In Bangladesh, Kenya, Tanzania, and the DRC, perceptions of social norms have positive and negative consequences, risk, severity, trust in institutions, safety, and expected access to COVID-19 vaccines have the highest associations with the acceptance of COVID-19 vaccines [6]. Meanwhile, in Myanmar and India, behavioral determinants, such as trust in COVID-19 information provided by leaders, religion, and perceived effectiveness of COVID-19 vaccines, are significantly related to COVID-19 vaccination intake [15].

In this paper, we analyze the factors that drive remote rural households to become vaccinated against COVID-19 when access is guaranteed. We surveyed a random sample of 800 households (1,592 individuals) from 22 municipalities in remote rural areas of Antioquia, Colombia. We find that four determinants increase the probability of having at least the first dose of the COVID-19 vaccine: access to information (measured by Internet access and satellite connection), trust in police and army, the perceived risk of contracting COVID-19 and the distance to the municipal capital. These results can contribute to developing effective vaccination campaigns for future health emergencies in remote rural areas.

This paper is organized as follows. *Introduction* section reviews the COVID-19 vaccination campaigns in rural Colombia. *Methods* section presents methods and data. *Results* section presents the results. *Discussion* section concludes.

### COVID-19 Vaccination Campaigns in Rural Colombia

The first batch of vaccines arrived in Colombia in February 2021 [16]; in July 2022, approximately 82.5% Colombians had at least one dose of the COVID-19 vaccine and around 70.5% were fully vaccinated [7]. To efficiently apply and distribute vaccines throughout the country, the Ministry of Health prioritized vaccine access according to a person’s risk, considering factors such as work, age, and underlying diseases [16, 17]. Vaccines were offered free of charge to ensure access for economically vulnerable populations and to achieve an equitable distribution [17]. To effectively vaccinate the population, the government designed campaigns that stressed the importance and efficiency of the vaccine to help restore safely to “normality”, [18].

The national government designed numerous campaigns to ensure the distribution of vaccines in all areas of the country. Thanks to an agreement between the regional government and private companies, vaccination posts were established in public transport [19] and some municipalities. For example, in Rionegro, drive-through vaccination stations allowed citizens to receive the vaccine in their cars [20]. Vaccine posts were established in schools or community action offices [17] for people who lived far from health centers. This strategy was intended to allow the entire Colombian population to access state-funded vaccines regardless of where they live. In addition, to increase vaccination rates, the regional government ran a public awareness campaign *Vacunarte te da más* (being vaccinated gives you more), encouraging its citizens to get vaccinated. This campaign offered discounts and additional benefits in some venues, such as movie theaters and shopping centers, to people who presented their vaccination cards [21].

## Methods

### Data

We conducted a regional multitopic household survey of 800 households (1,592 individuals) from 22 municipalities in rural Antioquia. This constitutes a representative sample for all rural sectors in Antioquia. Our survey collected information related to choices, perspectives, and concerns on six topics: land, ease of access to credit, health, household assets, welfare, and sociodemographic characteristics of the household. Furthermore, we collected a household-level roster, allowing us to construct household-level variables for respondents and the people living with them. We collected several variables, including age, education level, place of birth, and type of employment contract.

To analyze COVID-19 vaccination rates, we built two approaches:

• Share of household members with at least one dose

$$Atleastonedose=\frac{Numberofhouseholdmembersoverage14withatleastonedose}{Totalhouseholdmembersoverage14}$$

• Share of household members fully vaccinated (two doses)

$$FullyVaccinated=\frac{Numberofhouseholdmembersoverage14withmorethanonedose}{Totalhouseholdmembersoverage14}$$

### Descriptive Statistics

The average age of the head of household is 46.6 years, the average household size is 3.7 people, and an average dependency rate of 0.7%. Only 4.8% report having a woman as head of household. Regarding household assets, 93.1% of households report having at least one television at home, 92.4% have a refrigerator, 55.7% have a washing machine, 60.1% have access to drinking water, and 95.5% have access to electricity. There is low access to communication technologies, with only 22.6% of households reported having an internet connection, and 15.7% having a satellite connection. Respondents report relatively low levels of trust. We find that 53.1% of households say they do not trust the community action board, 81.4% say they do not trust the police or the armed forces, and 61.2% say they do trust the church. Most survey respondents (68.4%) say they are unhappy with health services.

Vaccination rates were relatively high, but did not reach the same number of households. By 20 February 2022, 76% of household members older than 14 years received at least one dose, and 55 percent received two doses. The analysis considers the share of household members who received at least one dose and were fully vaccinated. Table 1 describes the main characteristics of households with at least one dose of the COVID-19 vaccine. We find that at the time of the survey, households where the head of household has more than a middle school education report higher vaccination rates (4.1% vs. 2.1%). Households with internet access or satellite connection report higher vaccination rates for all household members older than 14, (2.41% vs. 1.79%) and (1.64% vs. 1.33%), respectively.

**TABLE 1:** Characteristics of the sample-at least one dose—Key Determinants of COVID-19 Vaccination Take-up in Remote Rural Areas: Evidence From Colombia (Antioquia, Colombia. 2021).

	(1)	(2)	(3)	(4)	(5)
	Mean	Mean if group: Less 100%	Mean if group: 100%	Difference in means	*p*-value
Panel A: General Household Characteristics					
Educational level: More than middle school	0.026	0.041	0.021	0.020	0.131
Quartile 1 of household wealth	0.244	0.327	0.218	0.109	0.002
Quartile 2 of household wealth	0.273	0.219	0.289	−0.070	0.055
Quartile 3 of household wealth	0.229	0.230	0.229	0.000	0.993
Quartile 4 of household wealth	0.254	0.224	0.263	−0.039	0.276
Age group: less 25	0.046	0.046	0.046	0.000	0.982
Age group: 26–50	0.552	0.658	0.519	0.139	0.001
Age group over 50	0.402	0.296	0.436	−0.140	0.000
Panel B: Household Services					
Do you have access to internet	0.226	0.179	0.241	−0.062	0.070
Satellite connection in your household	0.157	0.133	0.164	−0.032	0.290
Panel C: Trust					
Do you trust the community action board?	0.469	0.429	0.481	−0.053	0.198
Do you trust the church	0.612	0.638	0.603	0.035	0.389
Do you trust the armed forces and the police?	0.253	0.173	0.278	−0.105	0.003
Satisfied with: service-health center ?	0.316	0.342	0.307	0.035	0.366
Panel D: Information					
To get information use: Newspaper	0.064	0.077	0.060	0.016	0.416
To get information use: Radio or Tv	0.846	0.827	0.852	−0.026	0.390
To get information use: internet	0.427	0.459	0.416	0.043	0.291
To get information use: social media	0.455	0.454	0.455	−0.001	0.977
To get information use: Friend’s conversation	0.619	0.592	0.628	−0.036	0.369
Panel D: COVID-19					
Worried about: sick with COVID-19	0.613	0.510	0.646	−0.135	0.001
Worried about: your friends or family because COVID-19	0.703	0.612	0.732	−0.119	0.001
Have you known anyone who has died for COVID-19	0.451	0.459	0.449	0.010	0.799
Do you known anyone hospital-admitted for COVID-19	0.416	0.454	0.403	0.051	0.209

Note: Mean if group equal 100% refers to households in which all members over the age of 14 have at least one vaccine.

Sources: Gottingen University, EAFIT, and National University survey, Antioquia Colombia, 2021.

Households who report trusting the community action board, police, and the armed forces report higher vaccination rates (48.1% vs. 42.9%) and (27.8% vs. 17.3%), respectively. On the other hand, households who report trusting the church and those who report being satisfied with their health services center report lower vaccination rates (60.3% vs. 63.8%) and (30.7% vs. 34.2%), respectively.

A closer examination of the household survey indicates that households that obtain information from newspapers or on the Internet are on average less vaccinated (6.0% vs. 7.7%) and (41.6% vs. 45.9%). Households that receive information through radio or television, social networks, or conversations with friends are, on average more vaccinated, (85.2% vs. 82.7%), (45.5% vs. 45.4%), and (62.8% vs. 59.2%), respectively. Finally, households whose heads of household report being concerned about COVID-19 report higher vaccination rates (64.6%).

Table 2 describes the main characteristics of households with more than one dose of the COVID-19 vaccine (“fully vaccinated”). We find that households where the head of household has more than a middle school education have lower vaccination rates at the time of the survey (2.3% vs. 3.0%). Households with internet access and satellite connection have lower vaccination rates for all household members over age 14, (22.0% vs. 23.4%) and (14.9% vs. 16.8%), respectively.

**TABLE 2:** Characteristics of the sample: Total vaccination—Key Determinants of COVID-19 Vaccination Take-up in Remote Rural Areas: Evidence From Colombia (Antioquia, Colombia. 2021).

	(1)	(2)	(3)	(4)	(5)
	Mean	Mean if group: Less 100%	Mean if group: 100%	Difference in means	*p*-value
Panel A: General Characteristics					
Educational level: More than middle school	0.026	0.030	0.023	0.007	0.544
Quartile 1 of household wealth	0.244	0.290	0.212	0.079	0.010
Quartile 2 of household wealth	0.273	0.275	0.270	0.005	0.875
Quartile 3 of household wealth	0.229	0.198	0.252	−0.054	0.072
Quartile 4 of household wealth	0.254	0.237	0.266	−0.030	0.339
Age group: less 25	0.046	0.042	0.048	−0.006	0.673
Age group: 26–50	0.552	0.629	0.499	0.130	0.000
Age group: over 50	0.402	0.329	0.453	−0.123	0.000
Panel B: Household Services					
Do you have access to internet	0.226	0.234	0.220	0.013	0.654
Satellite connection in your household	0.157	0.168	0.149	0.019	0.469
Panel C: Trust					
Do you trust the community action board?	0.469	0.425	0.499	−0.074	0.038
Do you trust the church	0.612	0.605	0.616	−0.012	0.740
Do you trust the armed forces and the police?	0.253	0.210	0.283	−0.073	0.018
Satisfied with: service-health center ?	0.316	0.344	0.296	0.049	0.142
Panel D: Information					
To get information use: Newspaper	0.064	0.075	0.057	0.018	0.297
To get information use:Radio or Tv	0.846	0.832	0.855	−0.023	0.372
To get information use: internet	0.427	0.410	0.438	−0.028	0.429
To get information use: Social media	0.455	0.428	0.474	−0.046	0.199
To get information use: Friend’s conversation	0.619	0.572	0.652	−0.080	0.021
Panel E: COVID-19					
Worried about: getting sick with COVID-19	0.613	0.554	0.654	−0.100	0.004
Worried about: your friends or family because COVID-19	0.703	0.653	0.738	−0.085	0.009
Have you known anyone who has died for COVID-19?	0.451	0.458	0.447	0.012	0.745
Have you known anyone hospital-admitted for COVID-19?	0.416	0.428	0.407	0.021	0.543

Note: mean if group equal 100% refers to households in which all members over the age of 14 have two doses of COVID-19 vaccine.

Sources: Gottingen University, EAFIT, and National University survey, Antioquia Colombia, 2021.

When we examine trust in institutions, we find that households who report trusting the community action board, the church, and the police and the armed forces are on average, more vaccinated (49.9% vs. 42.5%), (61.6% vs. 60.5%) and (28.3% vs. 21.0%), respectively. However, households who report being satisfied with their health services center are on average, less vaccinated (29.6% vs. 34.4%).

Furthermore, we find that households that obtain information through radio or television, the Internet, social networks, and conversations with friends are on average more vaccinated (85.5% vs. 83.2%), (43.8% vs. 41.0%) (47.4% vs. 42.8%), and (65.2% vs. 57.2%), respectively. Households who obtain information from newspapers are on average less vaccinated (5.7% vs. 7.5%). Finally, households whose heads of household said they were concerned about contracting COVID-19 report higher vaccination rates (65.4% vs. 55.4%).

### Empirical Framework

We estimate the main determinants of remote rural households becoming vaccinated once the availability of the vaccine was guaranteed. We define the linear probability model as follows:

$$Vac_{i}=\beta_{0}+\beta_{1}HH_{i}+\beta_{2}Wealth_{i}+\beta_{3}Remoteness_{i}+\beta_{4}Trust_{i}+\beta_{5}Risk_{i}+\gamma_{i}+\mu_{i}$$ (1)

The subscripts denote the household *i*. *Vac*_*i*_ is a vector that contains at least one dose of the COVID-19 vaccine and total vaccination; *HH* is a vector that contains household characteristics such as sex and education of the head of household, the number of children in the household, the employment rate and the dependency ratio. *Wealth*_*i*_ is a vector that contains a wealth index per household, as well as access to the Internet, a satellite connection, a smartphone, and a computer; *Remoteness*_*i*_ indicates the distance between the household *i* and the municipal capital; *Trust*_*i*_ is a vector that contains the levels of confidence in the community action board, the church, the police and the armed forces, and sentiment about the health services center; *Risk*_*i*_ is a vector that includes related variables, along with perception of risk about COVID-19; *γ*_*i*_ is a vector of controls that includes fixed effects of stratum and age of head of household; *μ*_*i*_ is an error term with mean zero corrected by sampling design.

## Results

### Major Determinants of Vaccination Take-Up: At Least One Dose

**FIGURE 1:**
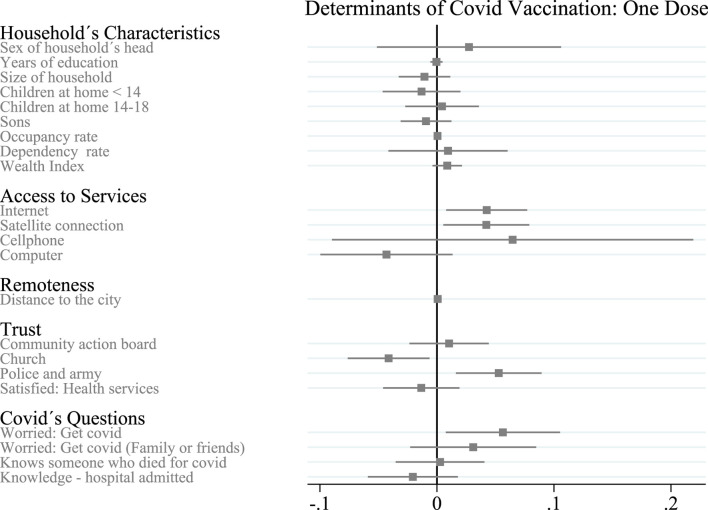
Determinants of vaccination take up: at least one dose in rural Colombia—Key Determinants of COVID-19 Vaccination Take-up in Remote Rural Areas: Evidence From Colombia (Antioquia, Colombia. 2021). Notes: The dependent variable is the average of household members over age 14 who had at least one dose of COVID-19 vaccine administered during the survey. Gray bars indicate 90% confidence intervals.

In this section, we discuss the main determinants of participation in vaccination for at least one dose. Table 3 and Figure 1 show how the results can change when we add the controls used in Eq. 1 one by one.

**TABLE 3:** Determinants of COVID-19 vaccination: at least one dose—Key Determinants of COVID-19 Vaccination Take-up in Remote Rural Areas: Evidence From Colombia (Antioquia, Colombia. 2021).

	(1)	(2)	(3)	(4)	(5)
	Model 1	Model 2	Model 3	Model 4	Model 5
Male	0.027	0.020	0.020	0.021	0.025
	(0.048)	(0.048)	(0.048)	(0.048)	(0.047)
Educational level: More than middle school (10th–13th)	−0.000	−0.001	−0.001	−0.000	0.001
	(0.003)	(0.003)	(0.003)	(0.003)	(0.003)
Household Size	−0.011	−0.009	−0.008	−0.008	−0.008
	(0.013)	(0.014)	(0.013)	(0.013)	(0.014)
Children under 14 years	−0.013	−0.014	−0.016	−0.015	−0.014
	(0.020)	(0.020)	(0.020)	(0.020)	(0.021)
Young people between 14 and 18 years old	0.004	0.005	0.007	0.007	0.007
	(0.019)	(0.019)	(0.019)	(0.019)	(0.019)
Sons and daughters	−0.009	−0.010	−0.012	−0.013	−0.012
	(0.013)	(0.013)	(0.013)	(0.013)	(0.014)
Percentage of people working	0.000	0.000	−0.000	−0.000	0.000
	(0.001)	(0.001)	(0.001)	(0.001)	(0.001)
Older than 60 years	0.009	0.006	−0.001	0.001	0.007
	(0.031)	(0.032)	(0.032)	(0.032)	(0.032)
Wealth index	0.009	0.010	0.012	0.011	
	(0.008)	(0.008)	(0.008)	(0.008)	
Do you have access to internet	0.043**	0.044**	0.043**	0.039*	
	(0.021)	(0.021)	(0.021)	(0.021)	
Do you have TV satelital connection in your household	0.042*	0.037*	0.039*	0.044**	
	(0.022)	(0.022)	(0.022)	(0.022)	
Do you have cellphone in your household	0.065	0.041	0.044	0.043	
	(0.094)	(0.093)	(0.093)	(0.093)	
Do you have computer in your household	−0.043	−0.050	−0.046	−0.047	
	(0.034)	(0.034)	(0.034)	(0.034)	
Distance in kilometres	0.001**	0.001*	0.001*		
	(0.000)	(0.000)	(0.000)		
Do you trust: Community Action Board	0.010	0.010			
	(0.021)	(0.021)			
Do you trust: Church	−0.041*	−0.035*			
	(0.021)	(0.021)			
Do you trust: Army or police	0.053**	0.051**			
	(0.022)	(0.023)			
Are you satisfied with the health system	−0.013	−0.018			
	(0.020)	(0.020)			
Are you worried about: sick with coronavirus	0.056*				
	(0.030)				
Worried about: friends or your family because COVID-19	0.031				
	(0.033)				
Have you Known anyone who has died for COVID-19	0.003				
	(0.023)				
Do you know anyone hospitalized for COVID-19	−0.021				
	(0.023)				
Head of household: Age	0.009	0.009	0.009	0.010	0.011
	(0.007)	(0.007)	(0.007)	(0.007)	(0.007)
Head of household: Age2	−0.000	−0.000	−0.000	−0.000	−0.000
	(0.000)	(0.000)	(0.000)	(0.000)	(0.000)
Stratum	0.048**	0.052***	0.053***	0.049***	0.050***
	(0.019)	(0.019)	(0.019)	(0.018)	(0.018)
Constant	0.450**	0.557***	0.554***	0.568***	0.555***
	(0.206)	(0.204)	(0.202)	(0.203)	(0.174)
Observations	811	811	811	811	811
R-squared	0.105	0.083	0.074	0.071	0.058

Robust standard errors in parentheses ***p < 0.01, **p < 0.05, *p < 0.1.

Dependent variable = totalvac_14/age_14 where totalvac_14 are people who have one or more vaccines and age over 14 and age_14 are people who are over 14.

#### Household Characteristics

The vector of household characteristics includes the gender of the head of household. This dummy variable defines whether the head of household has more than a middle school education, the size of the household, the number of children under age 14 in the household, the number of household members aged 14 to 18, the number of children, the percentage of household members who work, the number of people over age 60 and the household wealth index.

If the head of household is male, the likelihood that household members are vaccinated increases, at least with one dose (0.027). The household size decreases the probability that the household members are vaccinated with at least one dose (0.011). Households with children under 14 years of age and with children, in general, are more likely to be vaccinated, while having young people (between 14 and 18 years of age) in the household decreases the probability of being immunized. Households with people over 60 are more likely to have vaccinated members; a higher wealth index also increases the likelihood of vaccination. None of these results were statistically significant (90% CI) (Figure 1).

#### Access to Services

The vector of access to services includes access to the Internet, access to a satellite connection, having at least one smartphone per household, and having at least one computer per household. We find that access to the Internet increases the probability of being vaccinated (0.043); this result is statistically significant (95% CI). Access to a satellite connection shows a similar significant effect (0.042; 90% CI). Having at least one smartphone per household increases the likelihood that a household is vaccinated, but it is not statistically significant. Having at least one computer per household *decreases* the likelihood of being vaccinated, but this result is also not statistically significant.

#### Remoteness

The distance between home and the nearest municipal seat positively affects the probability of vaccination at home (0.001); this result is statistically significant (90% CI).

#### Confidence

The vector of trust includes confidence in the community action board, the church, the police and the army, and the level of satisfaction with the health system. We find that households that trust the community action board are more likely to be vaccinated. The level of satisfaction with the health system negatively affects the probability of being vaccinated. These results are not statistically significant. Trusting in the church decreases the probability of being vaccinated (0.041), while trusting in the police and the army increases the probability of being vaccinated (0.053). Both results are statistically significant (90% and 95% CI, respectively).

#### Perceptions of Risk of Contracting COVID-19

The vector of COVID-19 risk questions contains dummy variables that identify whether the head of household is concerned about getting COVID-19, whether the head of household is concerned that their friends or family may catch COVID-19, whether the head of household knows someone who has been hospitalized for COVID-19, and whether the head of household knows someone who has died from COVID-19. We find that households where the head of household is concerned about getting COVID-19 are more likely to be vaccinated (0.056); this result is statistically significant (90% CI). Households where the head of household is concerned that their family or friends may catch COVID-19 and where the head of household knows someone who has died from COVID-19, are more likely to be vaccinated, but households where the head of household knows someone who has been hospitalized from COVID-19 are less likely to be vaccinated. These results are not statistically significant.

### Main Determinants of Vaccination Take-Up: Fully Vaccinated

**FIGURE 2:**
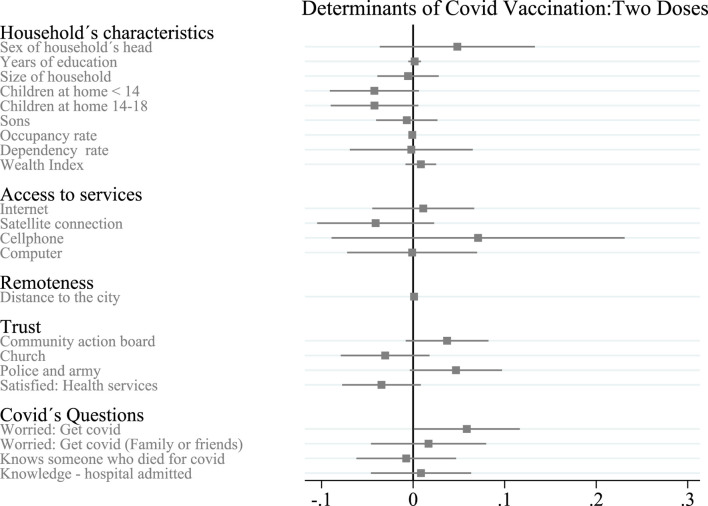
Determinants of covid vaccination in rural Antioquia for two doses—Key Determinants of COVID-19 Vaccination Take-up in Remote Rural Areas: Evidence From Colombia (Antioquia, Colombia. 2023). Notes: The dependent variable is the average of household members over age 14 who had two doses of COVID-19 vaccine administered at the time of the survey. Grey bars indicate 90% confidence intervals.

In this section, we discuss the main determinants of vaccination take-up for two doses (fully vaccinated), using the results of Eq. 1. Table 4 and Figure 2 show how the results can change when we add the controls used in Eq. 1 one by one.

**TABLE 4:** Determinants of COVID-19 vaccination: two doses—Key Determinants of COVID-19 Vaccination Take-up in Remote Rural Areas: Evidence From Colombia (Antioquia, Colombia. 2021).

	(1)	(2)	(3)	(4)	(5)
	Model 1	Model 2	Model 3	Model 4	Model 5
Male	0.048	0.036	0.041	0.043	0.049
	(0.051)	(0.050)	(0.051)	(0.052)	(0.051)
Educational level: More than middle school (10th–13th)	0.002	0.002	0.002	0.002	0.002
	(0.004)	(0.004)	(0.004)	(0.004)	(0.004)
Household Size	−0.006	−0.003	−0.003	−0.004	−0.004
	(0.020)	(0.020)	(0.020)	(0.020)	(0.020)
Children under 14 years	−0.042	−0.045	−0.046	−0.046	−0.049*
	(0.030)	(0.029)	(0.029)	(0.030)	(0.029)
Young people between 14 and 18 years old	−0.042	−0.043	−0.042	−0.042	−0.042
	(0.029)	(0.029)	(0.029)	(0.029)	(0.029)
Sons and daughters	−0.007	−0.008	−0.008	−0.009	−0.006
	(0.020)	(0.020)	(0.020)	(0.020)	(0.020)
Percentage of people working	−0.001	−0.001	−0.001*	−0.001**	−0.001**
	(0.001)	(0.001)	(0.001)	(0.001)	(0.001)
Older than 60years	−0.002	−0.007	−0.016	−0.012	−0.012
	(0.041)	(0.041)	(0.041)	(0.041)	(0.041)
Wealth index	0.008	0.011	0.013	0.011	
	(0.010)	(0.010)	(0.010)	(0.010)	
Do you have internet access?	0.011	0.013	0.012	0.005	
	(0.034)	(0.033)	(0.033)	(0.033)	
Do you have a satellite TV connection in your household	−0.041	−0.044	−0.042	−0.034	
	(0.039)	(0.038)	(0.038)	(0.038)	
Do you have cellphone in your household	0.071	0.053	0.058	0.056	
	(0.097)	(0.097)	(0.096)	(0.098)	
Do you have a computer in your house	−0.001	−0.007	−0.008	−0.009	
	(0.043)	(0.043)	(0.043)	(0.043)	
Distance in kilometres	0.001**	0.001**	0.001**		
	(0.000)	(0.000)	(0.000)		
Do you trust: Community Action Board	0.037	0.037			
	(0.027)	(0.027)			
Do you trust: Church	−0.031	−0.024			
	(0.029)	(0.029)			
Do you trust: Army or police	0.047	0.045			
	(0.031)	(0.031)			
Are you satisfied with the health system	−0.035	−0.039			
	(0.026)	(0.026)			
Are you worried about: being sick with coronavirus?	0.059*				
	(0.035)				
Worried about:friends or your family because COVID-19	0.017				
	(0.038)				
Have you Known anyone who has died for COVID-19?	−0.007				
	(0.033)				
Do you know anyone hospitalized for COVID-19	0.009				
	(0.033)				
Head of household: Age	0.005	0.005	0.006	0.007	0.007
	(0.008)	(0.008)	(0.008)	(0.008)	(0.008)
Head of household: Age2	−0.000	−0.000	−0.000	−0.000	−0.000
	(0.000)	(0.000)	(0.000)	(0.000)	(0.000)
Estrato	0.073***	0.077***	0.075***	0.068***	0.072***
	(0.025)	(0.025)	(0.025)	(0.025)	(0.024)
Constant	0.507**	0.592***	0.579**	0.602***	0.612***
	(0.226)	(0.223)	(0.225)	(0.226)	(0.203)
Observations	811	811	811	811	811
R-squared	0.093	0.083	0.074	0.070	0.067

Robust standard errors in parentheses ***p < 0.01, **p < 0.05, *p < 0.1.

Dependent variable = totalvac_14/age_14 where totalvac_14 are people who have two vaccines and are over 14 years old and age_14 are people over 14.

#### Household Characteristics

If the head of household is male, the probability of fully vaccinating household members increases. The higher the level of education of the head of the household, the greater the likelihood of full vaccination among the household members. Meanwhile, in a larger household, members are less likely to be vaccinated. The number of children under 14, children aged 14 to 18, the percentage of people working, and the number of people over 60 years in the household all decrease the probability that family members are fully vaccinated. A higher wealth index increases the probability of being fully vaccinated. None of these results was statistically significant (90% CI) (Figure 2).

#### Access to Services

Access to the Internet and having at least one cellphone per household both increase the probability of being fully vaccinated, while access to a satellite connection and having at least one computer per household both decrease the probability of being fully vaccinated; these results are not statistically significant.

#### Remoteness

The distance between home and the nearest municipal office increases the probability that family members are fully vaccinated (0.001); this result is statistically significant (95% CI).

#### Trust in Institutions

Households who trust the community action board, the police and the army are more likely to be fully vaccinated, while higher levels of satisfaction with the health system and higher levels of trust in the church decrease the probability that family members are fully vaccinated. These results are not statistically significant.

#### Perceptions of Risk of Contracting COVID-19

Households in which the head of household is concerned about getting COVID-19 are more likely to be fully vaccinated (0.059); this result is statistically significant (90% CI). Households in which the head of household was concerned about their family or friends catching COVID-19 or households in which the head of household knows someone hospitalized from COVID-19 are also more likely to be fully vaccinated. On the other hand, homes where the head of household knows someone who has died of COVID-19 are less likely to be fully vaccinated. These results are not statistically significant.

## Discussion

Our data suggest that four main determinants explain the variation in vaccine acceptance in some rural households in Antioquia, Colombia. We found that, on average, households with more access to information (measured by internet access and satellite connection) were more likely to receive the first dose of the COVID-19 vaccine. Specifically, we find that households without an internet connection were more likely to receive the first dose (0.043); this result is statistically significant (95% CI). Similarly, we find a similar result for households with satellite connections (0.042); this result is also statistically significant (90% CI) (Figure 1).

When we analyzed the role of information in the take-up of the second dose, we found no statistically significant coefficients for internet access or satellite connection for households (Figure 2). With these results, we can conclude that information access is a determinant for the take-up of the first dose but not necessarily for the second dose.

We also find that trust in institutions could influence household decisions about COVID-19 vaccination. Like Viswan et al. (2021) and Davis et al. (2022), we find that households that trust the government (measured by confidence in the police and the army) were more likely to receive the first dose (0.053); this result is statistically significant (95% CI). On the other hand, households that trust the church were less likely to receive the first dose (Figure 1). However, about the second dose, we did not find a statistically significant coefficient for any result related to trust in institutions. Furthermore, other measures of trust in institutions, such as trust in the community action board or satisfaction with health services, do not influence households’ decision to vaccinate, neither for the first nor the second dose.

Finally, like Viswan et al. (2021), our results suggest that households that felt more susceptible to COVID-19 (measured by concern about contracting COVID-19) were more likely to receive the first and second dose, (0.056) and (0.059), respectively. Both coefficients are statistically significant (90% CI) (Figures 1, 2).

Although the national government-funded vaccination campaign ensured that all households had access to the vaccine, evidence suggests that the campaign had a greater impact on the first dose: 75.8% of the households in the sample reported that 100% of their members over 14 years of age received the first dose, compared to just 58.8% of households for the second dose. Considering that both doses were available to the entire population, we can conclude that access to vaccines was no longer a determining factor for the acceptance of household vaccinations, at least for 17.0% of our sample.

We find that access to information (measured by internet access and satellite connection), trust in police and army, the perceived risk of contracting COVID-19, and the distance to the municipal capital are all determinants that increase the probability of having at least the first dose. However, trust in the church decreases the probability at the household level.

When we talk about the second dose, the importance of access to information, trust in the police and the army, and trust in the church all lose importance. The only two determinants that increase the probability of fully vaccinating are distance from the municipal capital and the perceived risk of contracting COVID-19. We hypothesize that distance to the municipal capital increases the probability of vaccination because of the campaigns carried out by the national government, which set up nonconventional vaccination sites such as schools and community action board offices. These sites gave the entire population access to the vaccine, even in the country's most remote areas.

### Implications for Policy

The willingness to vaccinate is crucial, especially in a global health issue where the only viable solution is to achieve “herd immunity,” which can only be attained through widespread vaccination. This importance is further accentuated in areas where people often face greater economic challenges than the national average, such as rural areas, where the ability to cope with diseases may be limited. In this scenario, more research is needed to address the various factors influencing people’s willingness to vaccinate, especially in rural settings.

This study explored the importance of communication, measured by internet access and satellite connections, in people’s willingness to vaccinate. Therefore, public health investments related to vaccination should consider the availability of vaccines and the places where they are intended to be taken. In other words, it is essential to consider that in rural areas, the likelihood of accessing information through “traditional” media decreases as households move away from urban areas. Therefore, it is necessary to design campaigns that allow households in very remote areas of the capital to stay informed.

Furthermore, it was found that trust in the army and police is a key determinant that leads households to increase their probability of vaccination. Given this, it is important to consider again that it is crucial to have the supply or availability of the vaccine and trust the provider. In rural areas, where the army or police may represent the presence of the state, these figures play an important role in deciding to get vaccinated. Similarly, this occurs with the church, where belonging to a church decreases the likelihood that households are vaccinated. This underscores the ultimate need to include relevant groups or individuals who have representative roles within communities in vaccination campaigns.

### Strengths and Limitations

We assume that by addressing the preferences or decisions of the households regarding vaccination, we indirectly measure the demand for vaccines from these households. However, it is essential to recognize that the actual availability of vaccines in each municipality is a factor that we cannot directly measure. Although we have reports and figures provided by the national government on vaccine distribution and administration, the quality and periodicity of this information may limit our complete understanding of vaccine supply, especially in rural areas of the country.

It is also crucial to acknowledge that the findings derived from this research are only related to the household level. Therefore, no possible behavioral changes can be observed that may occur within a household, impacting an individual’s inclination toward vaccination. This includes scenarios where individuals coexist with varying vaccination rates. So, future research prioritize examining the dynamics or disparities that may arise in individuals’ willingness to vaccinate within the same household.
